# The Battle and the Burthens of Life

**Published:** 1854-07

**Authors:** 


### THE BATTLE AND THE BURTHENS OF LIFE.

It is an admitted fact that less than one half of the human family have to toil like galley-slaves to support "the rest of mankind" in idleness. Every State is burthened with its almshouses; and every family with its hangers-on, whose only function is to eat what others earn—to consume what others produce. They are either too weak, or too proud, or too lazy to labor; and consequently these miserable drones must be supported by the industrious bees who supply all the honey for the human hive.

It is a pity that the stern justice of that good old Bible ordinance: "He that will not work, neither shall he eat," were not more rigorously applied to that innumerable army of Do-No-things, who, like the frogs of Egypt, infest all the avenues of civilized society. The man who consumes more than he earns is guilty of robbery. He who hangs a mere clog upon the social state, has no legitimate right to the food he swallows, or to the clothes he wears. Every dollar he spends is a fraud upon his toiling neighbor; and the sooner he vanishes from existence, and gives place to a man with work in him, the better will it be for the town, village or hamlet which his good-for-nothing body encumbers. The idea that a portion of humanity is made of porcelain, and not of common clay—for ornament and not for use, may do for the creed of dandies, who saunter through life, basking in the social sunshine which they have never helped to create; but it must be scouted by all honest men who get their bread by the sweat of their brows, and by lives of honorable toil fulfil the fiat of their Creator.

In this city of half a million of human beings, how large a portion dawdle away existence in idleness and sloth; or contrive by trading, shaving, gambling, and stealing, to fill their mouths and their pockets with the fruits of other men's labors! To eat, sleep, and show themselves; and to jolt their jaded sensibilities into some faint semblance of pleasurable emotions is the grand problem of a great majority of those who count in the census, but who form no part of the element that "constitutes a State;" while the toiling few are struggling in fields, in marts, in workshops, and on 'change, to produce the wherewithal to satisfy the appetites and the ambitions of the lazy, loafing masses.

And is there no relief from this insupportable burden? Will society always compel one willing worker to supply the mouths of half a dozen idlers? Shall one sturdy fellow always sweat at the oar, rowing a whole boat-load of indolent passengers across the ferry of Life? Must one poor man always turn the wheel that keeps the social machinery in motion?—No, not when a proper estimate is bestowed upon the dignity of labor—when a just reprobation is visited upon the infamy—the crime of idleness. When society shall take off its hat, and make obeisance to the swart workers who feed and clothe it, and point with the finger of scorn at the effeminate drones who consume it, *then* toil will be justly honored; while idleness, pauperism, beggary, and crime, in all their forms, will be crowded into the limbos of outer darkness where they belong.—*New York Mirror.*

